# Editorial: Genomics of Experimental Evolution

**DOI:** 10.3389/fgene.2017.00093

**Published:** 2017-09-12

**Authors:** Lee F. Greer

**Affiliations:** Network for Experimental Research on Evolution, Department of Ecology and Evolutionary Biology, School of Biological Sciences, University of California, Irvine Irvine, CA, United States

**Keywords:** experimental evolution, experimental genomics, genomics of domestication, high throughput genomics, long term experimental evolution, adaptation in experimental in long term experimental evolution

Biology as a science is beginning its third century with new genomic foundations. The challenge of combining experimental evolution with genomics, is being met by a growing number of researchers, including the contributors to the current Frontier Topics Issue volume on the Genomics of Experimental Evolution, who in part discuss emerging new strategies. Today, experimental evolutionary genomic studies have the promise and perhaps the possibility of showing causal connections within the vast assemblages of genomic data generated.

Although experimental evolution has in a sense been conducted since animal and crop domestication, an early joining of experimental evolution with genetics was a classic mid-twentieth century work on selection and genetic drift in fruitflies (Dobzhansky and Pavlovsky, 1957). An early application of genomics to experimental evolution was conducted on yeast, the application of microarray gene expression assays (Sniegowski, 1999). The foundations for future experimental evolutionary genomics studies were laid by multiple-replicated, long-term experiments starting as early as 1980 in fruitflies (Rose, 1984; Rose et al., 2004) and in 1988 in *E. coli* (Lenski et al., 1991; Lenski and Travisano, 1994; http://myxo.css.msu.edu/ecoli/; see also Tenaillon et al., 2016). Genomic sequencing has since been applied to long term experiments both in bacteria (Barrick et al., 2009) and in fruitflies (Burke et al., 2010), with a proliferation of similar studies. Such evolve and resequence studies (Turner et al., 2011) have had a measure of success along with challenges, subjecting experimental evolution cohorts to full high throughput genome sequencing and analysis (Schlötterer et al., 2014a,b). Increasingly sophisticated methods are being explored to analyse adaptive footprints in full genomes (e.g., Topa et al., 2015), assaying of gene expression on the whole genome level (e.g., Chang et al., 2015), and integration of multiple genomic level data sets (e.g., Feugeas et al., 2016).

Our contributors have helped address this emerging field's challenges. The first topic paper (Pesko et al.) explores how in cell cultures experimental gene variant RNA viruses of the *Mononegavirales* order have gene order-dependent varying fitnesses within both immune compromised and non-compromised prostate cancer cell lines. Using a data re-analysis of published studies in microbial evolution as well as simulations, the second topic perspective paper, Couce and Tenaillon consider the basis for the recurring observation in microbial evolution that fitness adaptation rates decline with adaptation across bacterial, viral, and yeast evolution. This powerful observation stemming from experimental evolution may inform us at a fundamental level about the nature of the multi-dimensional adaptive Euclidean space models of adaptation. In our third topic paper, Matos et al. in their opinion piece discuss how the application of genome-wide techniques in one of the oldest experimental evolution model organisms, *Drosophila*, help us understand the contours and tendencies of evolution. In their original research, the fourth topic paper by Graves et al. explores a very different technical territory by introducing the implications of microbial life adaptation to the emerging nanotechnology of heavy metals and their oxides, engineered nanoparticles (eNPs)—specifically how rapidly *Escherichia coli* adapted to silver eNPs. Their findings have implications for the use of heavy metal eNPs as antimicrobials on targeted and natural populations of microbes. In the fifth topic paper, O'Rourke et al. consider operon-based gain-of-function mutations leading to colony morphology variation in biofilm and planktonic growth within the *Burkholderia cenocepacia* pathogen complex, and the implications for human and agricultural thriving. Deatherage et al. in the final topic paper, lay out the challenges to identifying structural variants (SVs) in microbial genome evolutionary studies, which are more technically difficult to detect than nucleotide polymorphisms (NPs) and insertion deletions (indels). They discuss the theory, sensitivity, and simulations in applying their *breseq* analysis pipeline to the detection of SVs.

## Author contributions

The author confirms being the sole contributor of this work and approved it for publication.

### Conflict of interest statement

The author declares that the research was conducted in the absence of any commercial or financial relationships that could be construed as a potential conflict of interest.

## References

[B1] Barrick J. E. Yu D. S. Yoon S. H. Jeong H. Oh T. K. Schneider D.. (2009). Genome evolution and adaptation in a long-term experiment with *Escherichia coli*. Nature 461, 1243–1247. 10.1038/nature08480 19838166

[B2] Burke M. K. Dunham J. P. Shahrestani P. Thornton K. R. Rose M. R. Long A. D. (2010). Genome-wide analysis of a long-term evolution experiment with *Drosophila*. Nature 467, 587–590. 10.1038/nature09352 20844486

[B3] Chang T. C. Pertea M. Lee S. Salzberg S. L. Mendell J. T. (2015). Genome-wide annotation of microRNA primary transcript structures reveals novel regulatory mechanisms. Genome Res. 25, 1401–1409. 10.1101/gr.193607.115 26290535 PMC4561498

[B4] Dobzhansky T. Pavlovsky O. (1957). An experimental study of interaction between genetic drift and natural selection. Evolution 11, 311–319. 10.2307/2405795

[B5] Feugeas J.-P. Tourret J. Launay A. Bouvet O. Hoede C. Denamur E.. (2016). Links between transcription, environmental adaptation and gene variability in *Escherichia coli*: correlations between gene expression and gene variability reflect growth efficiencies. Mol. Biol. Evol. 33, 2515–2529. 10.1093/molbev/msw105 27352853

[B6] Lenski R. E. Travisano M. (1994). Dynamics of adaptation and diversification: a 10,000-generation experiment with bacterial populations. Proc. Natl. Acad. Sci. U.S.A. 91, 6808–6814. [Reprinted in: Ayala, F. J., and Fitch, W. M. (eds.). (1995). *Tempo and Mode in Evolution: Genetics and Paleontology 50 Years after Simpson*. Washington, DC: National Academy Press. pp. 253–273]. 8041701 10.1073/pnas.91.15.6808 PMC44287

[B7] Lenski R. E. Rose M. R. Simpson S. C. Tadler S. C. (1991). Long-term experimental evolution in Escherichia coli. I. Adaptation and divergence during 2,000 generations. Am. Nat. 138, 1315–1341.

[B8] Rose M. R. (1984). Artificial selection on a fitness component in Drosophila melanogaster. Evolution 38, 516–526. 10.2307/2408701 28555975

[B9] Rose M. R. Passananti H. B. Matos M. (eds.). (2004). Methuselah flies: A Case Study in the Evolution of Aging. Singapore: World Scientific Publishing.

[B10] Schlötterer C. Kofler R. Versace E. Tobler R. Franssen S. U. (2014a). Combining experimental evolution with next-generation sequencing: a powerful tool to study adaptation from standing genetic variation. Heredity 114, 431–440. 10.1038/hdy.2014.86 25269380 PMC4815507

[B11] Schlötterer C. Tobler R. Kofler R. Nolte V. (2014b). Sequencing pools of individuals - mining genome-wide polymorphism data without big funding. Nat. Rev. Genet. 15, 749–763. 10.1038/nrg3803 25246196

[B12] Sniegowski P. (1999). The genomics of adaptation in yeast. Curr. Biol. 9, r897–r898. 10607555 10.1016/s0960-9822(00)80078-0

[B13] Tenaillon O. Barrick J. E. Ribeck N. Deatherage D. E. Blanchard J. L. Dasgupta A.. (2016). Tempo and mode of genome evolution in a 50,000-generation experiment. Nature 536, 165–170. 10.1038/nature18959 27479321 PMC4988878

[B14] Topa H. Jónás Á. Kofler R. Kosiol C. Honkela A. (2015). Gaussian process test for high-throughput sequencing time series: application to experimental evolution. Bioinformatics 31, 1762–1770. 10.1093/bioinformatics/btv014 25614471 PMC4443671

[B15] Turner T. L. Stewart A. D. Fields A. T. Rice W. R. Tarone A. M. (2011). Population-based resequencing of experimentally evolved populations reveals the genetic basis of body size variation in *Drosophila* melanogaster. PLoS Genet. 7:e1001336. 10.1371/journal.pgen.1001336 21437274 PMC3060078

